# Performance Analysis and Optimization of a Cooperative Transmission Protocol in NOMA-Assisted Cognitive Radio Networks with Discrete Energy Harvesting

**DOI:** 10.3390/e23060785

**Published:** 2021-06-20

**Authors:** Hui Wang, Ronghua Shi, Kun Tang, Jian Dong, Shaowei Liao

**Affiliations:** 1 School of Computer Science and Engineering, Central South University, No. 932 Lushannan Road, Changsha 410083, China; wanghui0517@csu.edu.cn (H.W.); shirh@csu.edu.cn (R.S.); 2 Guangdong Provincial Key Laboratory of Millimeter-Wave and Terahertz, School of Electronic and Information Engineering, South China University of Technology, No. 381 Wushan Road, Guangzhou 510640, China; liaoshaowei@scut.edu.cn; 3 Pazhou Lab, No. 248 Pazhouqiaotou Street, Guangzhou 510220, China

**Keywords:** cognitive radio network, NOMA, cooperative transmission, discrete energy harvesting, energy efficiency

## Abstract

In this paper, we propose a spectrum-sharing protocol for a cooperative cognitive radio network based on non-orthogonal multiple access technology, where the base station (BS) transmits the superimposed signal to the primary user and secondary user with/without the assistance of a relay station (RS) by adopting the decode-and-forward technique. RS performs discrete-time energy harvesting for opportunistically cooperative transmission. If the RS harvests sufficient energy, the system performs cooperative transmission; otherwise, the system performs direct transmission. Moreover, the outage probabilities and outage capacities of both primary and secondary systems are analyzed, and the corresponding closed-form expressions are derived. In addition, one optimization problem is formulated, where our objective is to maximize the energy efficiency of the secondary system while ensuring that of the primary system exceeds or equals a threshold value. A joint optimization algorithm of power allocation at BS and RS is considered to solve the optimization problem and to realize a mutual improvement in the performance of energy efficiency for both the primary and secondary systems. The simulation results demonstrate the validity of the analysis results and prove that the proposed transmission scheme has a higher energy efficiency than the direct transmission scheme and the transmission scheme with simultaneous wireless information and power transfer technology.

## 1. Introduction

With the promotion of 5G, Cognitive radio (CR) has gradually attracted researchers’ attention, which is regarded as an important technology to improve the spectrum utilization efficiency of 5G networks [1]. A cognitive radio is a wireless communication system that intelligently utilizes any available side information about the activity, channel conditions, codebooks, or messages of other nodes with which it shares the spectrum. The main purpose of CR is to realize dynamic spectrum access and sharing through an understanding of the surrounding environment and adjustment of operating parameters. CR detects the unused spectrum in the surrounding radio environment during the cognitive cycle and allocates the unused spectrum to low-priority secondary users in an opportunistic or cooperative manner [2]. There are three main CR networks, i.e., underlay, overlay and interweave, in which the overlay approach is widely used. In overlay systems, the CR uses complex signal processing and coding to maintain or improve the communication between nodes [3]. It allows primary users (PUs) and secondary users (SUs) to utilize the same frequency band for communication to realize effective spectrum utilizing. However, the interference from the SUs will affect the PU performance. To solve this problem, we combine non-orthogonal multiple access (NOMA) technology with CR. NOMA technology supports the simultaneous information transmission of multiple users under the same time-frequency resource by allocating different power domain levels [4,5]. The implementation process of NOMA employs power domain multiplexing for signal combination at transmitters and then utilizes the successive interference cancellation (SIC) technique to detect a signal at the receivers [6]. Therefore, the proper combination of NOMA and CR can reduce interference and make better use of spectrum resources. To date, there are three main cognitive NOMA architectures, namely, underlay NOMA network, overlay NOMA network, and CR-NOMA network [7,8]. Besides, the cooperative relay strategy is added to extend the transmission distance and reduce the interference within the network.

Moreover, in order to improve the system performance and prolong the life of sensor nodes, we introduce wireless energy harvesting (EH) technology for the relay node. The radio frequency (RF) signal sent by the transmitter can be regarded as an energy resource and the amount of harvested RF energy within a fixed distance is predictable and relatively stable over time, which can prolong the life of the network and provide great convenience to mobile users [9].

### 1.1. Related Work

According to the above analyses, an increasing number of researchers are focusing on the combination of NOMA and cognitive radio network (CRN). In [10], the authors proposed a NOMA-based CRN under the partial relay selection scheme, in which both *k* half-duplex technology and DF relaying can be used to assist the secondary base station to deliver information for SUs. In [11], the authors dealt with the long-term throughput maximization of an uplink NOMA in the CRN and a combination of NOMA and time division multiple access was proposed to reduce the complexity of massive wireless communication systems while a deep Q learning algorithm was employed to maximize the long-term throughput of the system. The authors in [12] studied the optimal power allocation problem of SU with NOMA in cognitive mobile radio network, which converted long term evolution and wireless fidelity into a heterogeneous primary mobile network. The authors of [13] investigated another optimal power allocation problem in the controllable range, considering the interference of the primary system of the non-orthogonal cognitive radio vehicular ad-hoc networks, where the mobile vehicle node borrowed the unused wireless spectrum belonging to the primary network and completed an information exchange with the assistance of other independent nodes. In the context of CRN and NOMA, EH is an effective way to prolong the life of sensor nodes. An distributed transmission power control mechanism for the CR sensor network with EH was proposed in [14], in which each node dynamically determined the increase or decrease in its transmission power according to its own available power and the available power of neighboring nodes. The authors in [15] studied the cooperative CRN with EH, under the constraints of the primary system performance and proposed an energy allocation ratio parameter to achieve the target throughput. In [16], the authors investigated a cognitive crossover network, where the SU’s energy-harvesting relay helped the primary user to transmit by using NOMA technology in the absence of a direct link. In [17], simultaneous wireless information and power transfer (SWIPT) technique was applied for NOMA-based cooperative CRN to provide a higher overall outage performance. In [18], the authors analyzed the performance of a NOMA-CR system, in which a multi-antenna full-duplex cognitive transmitter adopted NOMA technology to assist the primary transmitter with nonlinear EH to transmit the signal. The authors in [19,20] considered a CR-NOMA network with EH, which selected the optimal energy harvesting time and the power allocation of the secondary transmitter to achieve the maximum secondary throughput.

### 1.2. Contribution

The above papers focused on the combined application of CRN and NOMA technology, and some of them also discussed the wireless continuous-time EH technology, which is not suitable for some practical applications, with a certain threshold value of transmission power. Therefore, we consider the combination of a cooperative CRN based on NOMA and discrete-time EH technology, where the base station (BS) transmits the superimposed signal to PU and SU with/without the assistance of the relay station (RS) by adopting a decode-and-forward (DF) technique. RS performs discrete-time energy harvesting for opportunistically cooperative transmission. If the RS harvests sufficient energy, the system performs cooperative transmission; otherwise, the system performs direct transmission. Furthermore, the authors obtain the optimal parameters through joint optimization analysis in [21], such as EH duration, channel resource allocation and transmission power. Inspired by this paper, we formulate an optimization problem about energy efficiency and propose the corresponding joint optimization algorithm of power allocation at BS and RS for the optimization problem. The main contributions of this paper are summarized as follows:

- We investigate a CR-NOMA network model with discrete energy harvesting and propose the corresponding cooperative transmission protocol, in which RS performs discrete-time energy harvesting for opportunistically cooperative transmission, and BS transmits the superimposed signal to PU and secondary user SU with/without the assistance of RS by adopting a DF relaying technique;
- The processes of charging and discharging RS’s battery is simulated by utilizing the Markov chain (MC) model. Then, the state transition probabilities of RS’s battery are analyzed and closed-form expressions of outage probabilities and outage capacities for both the primary and secondary systems are derived, which are validated by Monte Carlo simulation;
- To ensure the performance of the primary system, we propose a corresponding joint parameter optimization algorithm to obtain the optimal power allocation ratio to optimize the secondary system’s energy efficiency. The simulation results demonstrate that the proposed transmission scheme has a higher energy efficiency than the direct transmission scheme and another transmission scheme with SWIPT.

The remainder of this paper is organized as follows: In Section 1, the communication system model is introduced in detail and the corresponding cooperative transmission protocol is proposed, based on a DF relaying technique. In Section 2, we analyze the discrete-time energy harvesting model first, and then derive the analytical expressions of the outage probabilities for both primary and secondary systems. In Section 3, a joint parameter optimization algorithm for power allocation in BS and RS is proposed. The simulations and conclusion are presented in Section 4 and Section 5, respectively.

## 2. System Model and Transmission Protocol

**Figure 1:**
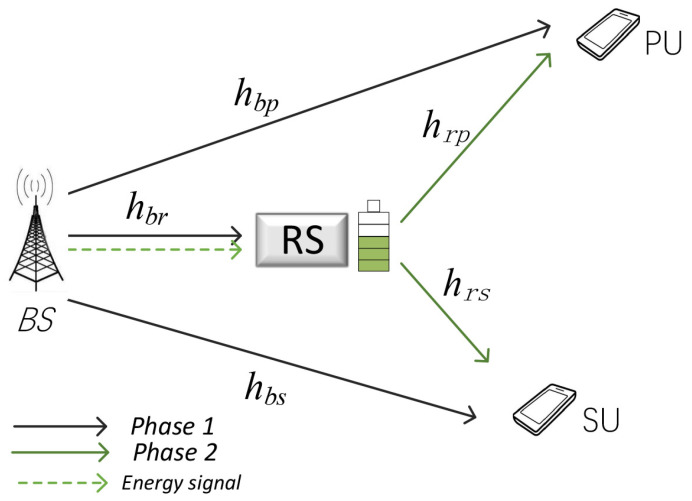
System Model.

As shown in Figure 1, we consider a NOMA-based cooperative CRN, where BS transmits information to PU and SU simultaneously through RS opportunistically relaying signals. In addition, the RS is equipped with a limited capacity battery to store the energy harvested from BS, and it can provide the opportunistic spectrum sharing service in the case of sufficient battery energy accumulation. We suppose that each node has been equipped with a single half-duplex antenna, and all channels in the system are the quasi-static Rayleigh fading channels [22]. $h_{bp}$, $h_{br}$, and $h_{bs}$ represent the channel coefficients between BS and PU, BS and RS, BS and SU, while $h_{rp}$ and $h_{rs}$ represent the channel coefficients between RS and PU, RS and SU, respectively. Therefore, channel gain can be expressed as $|h_{i}{|}^{2}\left(i=bp,br,bs,rp,rs\right)$, subject to exponential distribution with mean $\lambda_{i}$.

**Figure 2:**
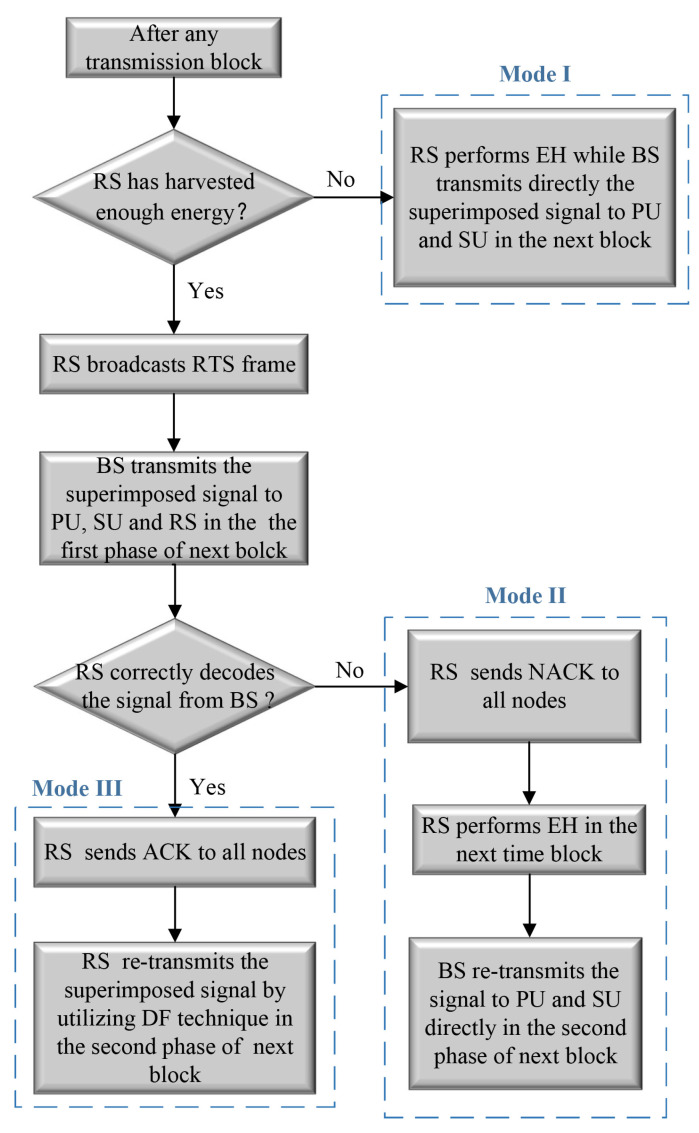
Flow Chart of proposed spectrum Scheme.

In the proposed model, the RS would calculate whether it has harvested enough energy to relay at the beginning of each time block. If not, the RS will perform energy-harvesting mode in the next time block, while the BS transmits information to both PU and SU directly. Otherwise, RS broadcasts a request to send (RTS), and the frame and cooperative transmission mode is carried out with DF technique in the next block. For cooperative transmission mode, we divide the transmission block into two equal-length phases. In the first phase, BS conveys a superimposed signal to PU, SU, and RS. In the second phase, RS first predicts whether the superimposed signal from BS can be successfully decoded. If RS could not decode the superimposed signal successfully, RS first broadcasts a negative acknowledgment (NACK) frame to all nodes and BS retransmits the superimposed signal to PU and SU, while RS continues to perform energy harvesting. If successful, the RS sends an acknowledgment (ACK) frame to all nodes first, and then recodes and retransmits the composited signal to both PU and SU, which will use the SIC technique to obtain their desired messages. Hence, there are three possible modes under the proposed relaying protocol, as shown in Figure 2.

When the RS’s battery’s energy has not reached the threshold value $E_{T}$, the system performs in energy harvesting mode, i.e., Mode I. Hence, the amount of harvested energy at RS in Mode I is given as

(1) $$E_{H}^{I}=\eta TP_{BS}{\left|h_{br}\right|}^{2}.$$

When the amount of harvested energy of RS reaches the threshold value $E_{T}$ the system performs in cooperative transmission mode. In the first phase, BS conveys a superimposed signal $x\left(t_{1}\right)=\sqrt{k_{1}}x_{p}+\sqrt{1-k_{1}}x_{s}$ to PU, SU and RS, where $x_{p}$ and $x_{s}$ represent required signals for PU and SU, respectively, and $k_{1}$ denotes power allocation coefficient at BS. The use of NOMA technology ensures that the transmission power of PU messages is always higher than that of SU messages, with $k_{1}>0.5$. The received signals at PU, SU and RS are expressed as

(2) $$\begin{array}{c} y_{p}\left(t_{1}\right)=\sqrt{P_{BS}}\left(h_{bp}\left(\sqrt{k_{1}}x_{p}+\sqrt{1-k_{1}}x_{s}\right)\right)+n_{bp},\\ y_{s}\left(t_{1}\right)=\sqrt{P_{BS}}\left(h_{bs}\left(\sqrt{k_{1}}x_{p}+\sqrt{1-k_{1}}x_{s}\right)\right)+n_{bs},\\ y_{r}\left(t_{1}\right)=\sqrt{P_{BS}}\left(h_{br}\left(\sqrt{k_{1}}x_{p}+\sqrt{1-k_{1}}x_{s}\right)\right)+n_{br}, \end{array}$$

respectively, where $P_{BS}$ represents a transmission power of BS and $n_{i}∼CN\left(0,\delta^{2}\right)\left(i=bp,bs,br\right)$ denotes the received additive white Gaussian noise (AWGN) at node PU, SU, and RS, respectively. Therefore, the received signal-to-interference-and-noise-ratio (SINR) at PU in the first transmission phase can be written as

(3) $$\gamma_{bp}=\frac{k_{1}P_{BS}{\left|h_{bp}\right|}^{2}}{\left(1-k_{1}\right)P_{BS}{\left|h_{bp}\right|}^{2}+\delta^{2}}.$$

SU applies the SIC technique to detect PU’s signal, and then cancels it to obtain its own signal. Hence, the SINRs for SU detecting the signals of PU and SU are given by

(4) $$\gamma_{bs1}=\frac{k_{1}P_{BS}{\left|h_{bs}\right|}^{2}}{\left(1-k_{1}\right)P_{BS}{\left|h_{bs}\right|}^{2}+\delta^{2}},$$

(5) $$\gamma_{bs2}=\frac{\left(1-k_{1}\right)P_{BS}{\left|h_{bs}\right|}^{2}}{\delta^{2}},$$

respectively.

Similar to SU, the SINRs for RS-detecting signals of PU and SU can be expressed as

(6) $$\gamma_{br1}=\frac{k_{1}P_{BS}{\left|h_{br}\right|}^{2}}{\left(1-k_{1}\right)P_{BS}{\left|h_{br}\right|}^{2}+\delta^{2}},$$

(7) $$\gamma_{br2}=\frac{\left(1-k_{1}\right)P_{BS}{\left|h_{br}\right|}^{2}}{\delta^{2}},$$

respectively.

Mode II means that the residual energy of RS’s battery is equals to or exceeds the threshold value $E_{T}$, but the superimposed signal is erroneously decoded at RS. Thus, BS retransmits a superimposed signal to PU and SU, while RS keeps energy harvesting. The amount of RS’s harvested energy in Mode II is same as Mode I, i.e., $E_{H}^{II}=E_{H}^{I}$. There are two cases which will lead to the emergence of Mode 2:

- Case 1: When the reachable rate of primary signal $x_{p}$ at the RS does not reach the primary target rate $r_{p}$, the RS cannot correctly detect the primary signal $x_{p}$. The case can be illustrated as
  (8) $$\log_{2}\left(1+\gamma_{br1}\right)<r_{p}.$$
- Case 2: When the reachable rate of primary signal $x_{p}$ at the RS reaches the primary target rate $r_{p}$, but the reachable rate of secondary signal $x_{s}$ at the RS is lower than the secondary target rate $r_{s}$, the case can be illustrated as
  (9) $$\begin{array}{c} \log_{2}\left(1+\gamma_{br1}\right)\geq r_{p}\\ \log_{2}\left(1+\gamma_{br2}\right)<r_{s}. \end{array}$$

In Mode III, RS has not only accumulated enough energy but also successfully decodes the superimposed signals. Then, RS will recode and retransmit the combined signal $x\left(t_{2}\right)=\sqrt{k_{2}}x_{p}+\sqrt{1-k_{2}}x_{s}$ to both SU and PU. During the second phase of cooperative transmission, because both PU and SU will cancel interference by using the signal decoded in the first phase, $k_{2}$ can be any value. Therefore, the signals observed at SU and PU in the second phase are derived as

(10) $$y_{p}\left(t_{2}\right)=\sqrt{P_{RS}}h_{rp}\left(\sqrt{k_{2}}x_{p}+\sqrt{1-k_{2}}x_{s}\right)+n_{rp},$$

(11) $$y_{s}\left(t_{2}\right)=\sqrt{P_{RS}}h_{rs}\left(\sqrt{k_{2}}x_{p}+\sqrt{1-k_{2}}x_{s}\right)+n_{rs},$$

respectively, where $k_{2}$ represents the power allocation coefficient at RS, $P_{RS}$ denotes the transmission power of RS, $n_{rp}∼CN\left(0,\delta^{2}\right),n_{rs}∼CN\left(0,\delta^{2}\right)$ are received AWGN at nodes PU and SU, respectively.

We assume that both PU and SU can subtract the other user’s information, received in the first transmission phase, to obtain their own desired message. Hence, the SINR for PU and SU can be, respectively, expressed as

(12) $$\gamma_{rp}=\frac{k_{2}P_{RS}{\left|h_{rp}\right|}^{2}}{\delta^{2}},$$

(13) $$\gamma_{rs}=\frac{\left(1-k_{2}\right)P_{RS}{\left|h_{rs}\right|}^{2}}{\delta^{2}}.$$

## 3. Analysis of Outage Performance

### 3.1. Energy Accumulation Analysis

A discrete time energy harvesting model [23] is adopted in this scheme. We assume that RS is equipped with a battery, which has finite capacity $E_{C}$ and is discretized into L+1 levels. Let $E_{l}\left(l=0,1,\ldots ,L\right)$ represent the quantization level defined by

(14) $$\begin{array}{cc} & E_{l}=\left[E_{l},E_{l+1}\right),0\leq l\leq L, \end{array}$$

where $E_{l}=\frac{lE_{C}}{L}$ is *l*th energy level of the battery. Therefore, we model RS’s charging and discharging behavior by adopting a Markov chain (MC) with L+1 states [23] and obtain further state transition probabilities. $S_{l}$ denotes a current energy level of RS and $P_{i,j}$ represents the transition probability from state $S_{i}$ to state $S_{j}$.

#### 3.1.1. $S_{0}\to S_{0}$

This scenario appears in Mode I when the energy $E_{H}^{I}$ harvested by the empty battery is less than $\frac{E_{C}}{L}$. Thus, the transition probability is given by

(15) $$\begin{array}{cc} P_{0,0} & =\mathrm{Pr}\left\{E_{H}^{I}<\frac{E_{C}}{L}\right\}=F_{{\left|h_{br}\right|}^{2}}\left(\frac{E_{C}}{L\eta P_{BS}}\right)\\ & =1-\exp(-\frac{E_{C}}{L\eta P_{BS}\lambda_{br}}) \end{array}$$

#### 3.1.2. $S_{0}\to S_{l}\left(0<l<L\right)$

The empty battery is partially charged and the amount of harvested energy falls between $E_{l}$ and $E_{l+1}$ in Mode I. Therefore, the transition probability can be derived as

(16) $$\begin{array}{cc} P_{0,l} & =\mathrm{Pr}\left\{\frac{lE_{C}}{L}<E_{H}^{I}<\frac{\left(l+1\right)E_{C}}{L}\right\}\\ & =F_{{\left|h_{br}\right|}^{2}}\left(\frac{\left(l+1\right)E_{C}}{L\eta P_{BS}}\right)-F_{{\left|h_{br}\right|}^{2}}\left(\frac{lE_{C}}{L\eta P_{BS}}\right) \end{array}$$

#### 3.1.3. $S_{0}\to S_{L}$

This case corresponds to situations where the empty battery is charged to full and the transition probability is evaluated as

(17) $$P_{0,L}=\mathrm{Pr}\left\{E_{H}^{I}\geq E_{C}\right\}=1-F_{{\left|h_{br}\right|}^{2}}\left(\frac{E_{C}}{\eta P_{BS}}\right)$$

#### 3.1.4. $S_{l}\to S_{l}\left(0<l<L\right)$

The status of RS’s battery with non-full energy remains unchanged when the harvested energy is less than $\frac{E_{C}}{L}$ in Mode I or Mode II. The transition probability is given as

(18) $$\begin{array}{cc} P_{l,l}= & \mathrm{Pr}\left\{\left[\left(E_{T}>\frac{lE_{C}}{L}\right)⋂\left(E_{H}^{I}<\frac{E_{C}}{L}\right)\right]\right.⋃\left[\left(E_{T}\leq \frac{lE_{C}}{L}\right)\right.\\ & ⋂\left.\left.\left(E_{H}^{II}<\frac{E_{C}}{L}\right)⋂\left(\left(\gamma_{br1}<R_{P}\right)⋃\left(\left(\gamma_{br1}\geq R_{P}\right)⋂\left.\left(\gamma_{br2}<R_{s}\right)\right)\right.\right)\right]\right\}\;\\ = & \left\{\begin{array}{c} F_{{\left|h_{br}\right|}^{2}}\left(\frac{E_{C}}{L\eta P_{BS}}\right),if\;E_{T}>\frac{lE_{C}}{L};\;\\ F_{{\left|h_{br}\right|}^{2}}\left(\varphi_{1}\right)F_{{\left|h_{br}\right|}^{2}}\left(\phi_{1}\right)-{F_{{\left|h_{br}\right|}^{2}}}^{2}\left(\varphi_{1}\right),\;\\ \;\;\;\;if\;E_{T}\leq \frac{lE_{C}}{L}\;and\;R_{P}\leq \frac{a_{1}}{c+a_{2}},R_{s}\leq \frac{a_{2}}{c};\;\\ F_{{\left|h_{br}\right|}^{2}}\left(\varphi_{1}\right)F_{{\left|h_{br}\right|}^{2}}\left(\frac{E_{C}}{L\eta P_{BS}}\right)-{F_{{\left|h_{br}\right|}^{2}}}^{2}\left(\varphi_{1}\right),\;\\ \;\;\;\;if\;E_{T}\leq \frac{lE_{C}}{L}\;and\;R_{P}\leq \frac{a_{1}}{c+a_{2}},R_{s}>\frac{a_{2}}{c};\;\\ F_{{\left|h_{br}\right|}^{2}}\left(\frac{E_{C}}{L\eta P_{BS}}\right)F_{{\left|h_{br}\right|}^{2}}\left(\phi_{1}\right)-F_{{\left|h_{br}\right|}^{2}}\left(\frac{E_{C}}{L\eta P_{BS}}\right)F_{{\left|h_{br}\right|}^{2}}\left(\varphi_{1}\right),\;\\ \;\;\;\;if\;E_{T}\leq \frac{lE_{C}}{L}\;and\;\frac{a_{1}}{c+a_{2}}\leq R_{P}\leq \frac{k_{1}}{1-k_{1}},R_{s}\leq \frac{a_{2}}{c};\;\\ {F_{{\left|h_{br}\right|}^{2}}}^{2}\left(\frac{E_{C}}{L\eta P_{BS}}\right)-F_{{\left|h_{br}\right|}^{2}}\left(\frac{E_{C}}{L\eta P_{BS}}\right)F_{{\left|h_{br}\right|}^{2}}\left(\varphi_{1}\right),\;\\ \;\;\;\;if\;E_{T}\leq \frac{lE_{C}}{L}\;and\;\frac{a_{1}}{c+a_{2}}\leq R_{P}\leq \frac{k_{1}}{1-k_{1}},R_{s}>\frac{a_{2}}{c};\;\\ 0,\;\;\;\;if\;E_{T}\leq \frac{lE_{C}}{L}andR_{P}>\frac{k_{1}}{1-k_{1}}.\; \end{array}\right. \end{array}$$

where $\varphi_{1}=\frac{R_{p}\delta^{2}}{P_{BS}\left(k_{1}-\left(1-k_{1}\right)R_{p}\right)}$, $\phi_{1}=\frac{R_{s}\delta^{2}}{P_{BS}\left(1-k_{1}\right)}$, $a_{1}=k_{1}E_{c}$, $a_{2}=\left(1-k_{1}\right)E_{c}$, $c=L\eta \delta^{2}$, $R_{p}=2^{r_{p}}-1$, $R_{s}=2^{r_{s}}-1$.

**Proof of (18).**  Please refer to Appendix A. □

#### 3.1.5. $S_{l}\to S_{m}\left(0<l<m<L\right)$

In Mode I or Mode II, the amount of energy harvested by the non-empty battery is between $E_{m-l}$ and $E_{m-l+1}$, and then the energy state of the battery will turn to level *m*. Therefore, its transition probability can be derived as -4.6cm0cm

(19) $$\begin{array}{cc} P_{l,m} & =\mathrm{Pr}\left\{\left[\left(E_{T}>\frac{lE_{C}}{L}\right)⋂\left(\frac{\left(m-l\right)E_{C}}{L}\leq E_{H}^{I}<\frac{\left(m-l+1\right)E_{C}}{L}\right)\right]⋃\left[\left(E_{T}\leq \frac{lE_{C}}{L}\right)\right.\right.\;\\ & \left.\left.⋂\left(\frac{\left(m-l\right)E_{C}}{L}\leq E_{H}^{II}<\frac{\left(m-l+1\right)E_{C}}{L}\right)⋂\left(\left(\gamma_{br1}<R_{P}\right)⋃\left(\left(\gamma_{br1}\geq R_{P}\right)⋂\left.\left(\gamma_{br2}<R_{s}\right)\right)\right.\right)\right]\right\}\;\\ & =\left\{\begin{array}{c} F_{{\left|h_{br}\right|}^{2}}\left(\frac{\left(m-l+1\right)E_{C}}{L\eta P_{BS}}\right)-F_{{\left|h_{br}\right|}^{2}}\left(\frac{\left(m-l\right)E_{C}}{L\eta P_{BS}}\right),if\;E_{T}>\frac{lE_{C}}{L};\;\\ 0,\;\;\;if\;E_{T}\leq \frac{lE_{C}}{L}\;and\;R_{P}<\frac{\left(m-l\right)a_{1}}{c+\left(m-l\right)a_{2}};\;\\ 0,\;\;\;ifE_{T}\leq \frac{lE_{C}}{L}and\frac{\left(m-l\right)a_{1}}{c+\left(m-l\right)a_{2}}\leq R_{P}\leq \frac{\left(m-l+1\right)a_{1}}{c+\left(m-l+1\right)a_{2}},R_{s}<\frac{\left(m-l\right)a_{2}}{c};\;\\ \left[F_{{\left|h_{br}\right|}^{2}}\left(\varphi_{1}\right)-F_{{\left|h_{br}\right|}^{2}}\left(\frac{\left(m-l\right)E_{C}}{L\eta P_{BS}}\right)\right]\left[F_{{\left|h_{br}\right|}^{2}}\left(\phi_{1}\right)-F_{{\left|h_{br}\right|}^{2}}\left(\varphi_{1}\right)\right],\;\\ \;\;\;if\;E_{T}\leq \frac{lE_{C}}{L}\;and\;\frac{\left(m-l\right)a_{1}}{c+\left(m-l\right)a_{2}}\leq R_{P}\leq \frac{\left(m-l+1\right)a_{1}}{c+\left(m-l+1\right)a_{2}},\frac{\left(m-l\right)a_{2}}{c}\leq R_{s}\leq \frac{\left(m-l+1\right)a_{2}}{c};\;\\ \left[F_{{\left|h_{br}\right|}^{2}}\left(\varphi_{1}\right)-F_{{\left|h_{br}\right|}^{2}}\left(\frac{\left(m-l\right)E_{C}}{L\eta P_{BS}}\right)\right]\left[F_{{\left|h_{br}\right|}^{2}}\left(\frac{\left(m-l+1\right)E_{C}}{L\eta P_{BS}}\right)-F_{{\left|h_{br}\right|}^{2}}\left(\varphi_{1}\right)\right]\;\\ \;\;\;\;if\;E_{T}\leq \frac{lE_{C}}{L}\;and\;\frac{\left(m-l\right)a_{1}}{c+\left(m-l\right)a_{2}}\leq R_{P}\leq \frac{\left(m-l+1\right)a_{1}}{c+\left(m-l+1\right)a_{2}},R_{s}>\frac{\left(m-l+1\right)a_{2}}{c};\;\\ 0,\;\;\;if\;E_{T}\leq \frac{lE_{C}}{L}\;and\;\frac{\left(m-l+1\right)a_{1}}{c+\left(m-l+1\right)a_{2}}<R_{P}<\frac{k_{1}}{1-k_{1}}. \end{array}\right. \end{array}$$

#### 3.1.6. $S_{l}\to S_{L}$

When the amount of harvested energy is equal to or exceeds $E_{L-l}$ in Mode I or Mode II, the non-empty battery will be charged to be full and its transition probability can be derived as

(20) $$\begin{array}{cc} P_{l,L} & =\mathrm{Pr}\left\{\left[\left(E_{T}>\frac{lE_{C}}{L}\right)⋂\left(E_{H}^{I}\geq \frac{\left(L-l\right)E_{C}}{L}\right)\right]\right.\;\\ & \;⋃[\left(E_{T}\leq \frac{lE_{C}}{L}\right)⋂\left(E_{H}^{II}\geq \frac{\left(L-l\right)E_{C}}{L}\right)\\ & \;\left.⋂\;\left(\;\left(\gamma_{br}^{x_{p}}<R_{P}\right)⋃\;\left(\;\left(\gamma_{br}^{x_{p}}\geq R_{P}\right)⋂\left.\left(\gamma_{br}^{x_{s}}<R_{s}\right)\right)\right.\right)]\;\right\}\\ = & \left\{\begin{array}{c} \;1-F_{{\left|h_{br}\right|}^{2}}\left(\frac{\left(L-l\right)E_{C}}{L\eta P_{BS}}\right),ifE_{T}>\frac{lE_{C}}{L};\;\\ \;0,\;\;\;if\;E_{T}\leq \frac{lE_{C}}{L}\;and\;R_{P}<\frac{\left(L-l\right)a_{1}}{c+\left(L-l\right)a_{2}}<\frac{k_{1}}{1-k_{1}};\;\\ \;0,\;\;\;if\;E_{T}\leq \frac{lE_{C}}{L}\;and\;\frac{\;\left(\;L-l\;\right)a_{1}}{c+\;\left(\;L-l\;\right)a_{2}}\leq R_{P}\;<\;\frac{k_{1}}{1-k_{1}},R_{s}\;\leq \;\frac{\;\left(\;L\;-\;l\;\right)a_{2}}{c};\;\\ \;\left[\;F_{{\;\left|h_{br}\;\right|}^{2}}\;\left(\;\varphi_{1}\;\right)\;-\;F_{{\left|h_{br}\right|}^{2}}\;\left(\;\frac{\;\left(\;L\;-\;l\right)\;E_{C}}{L\eta P_{BS}}\;\right)\;\right]\;\left[\;F_{{\left|h_{br}\right|}^{2}}\;\left(\;\phi_{1}\;\right)\;-\;F_{{\left|h_{br}\right|}^{2}}\;\left(\;\varphi_{1}\;\right)\;\right]\;,\;\\ \;\;\;\;if\;E_{T}\;\leq \;\frac{lE_{C}}{L}\;and\;\frac{\;\left(\;L\;-\;l\right)a_{1}}{c+\;\left(\;L\;-l\right)a_{2}}\;\leq \;R_{p}\;<\;\frac{k_{1}}{1-k_{1}},R_{s}\;>\;\frac{\;\left(\;L\;-\;l\right)a_{2}}{c};\;\\ \;0,\;\;\;if\;E_{T}\leq \frac{lE_{C}}{L}\;and\;R_{P}\geq \frac{k_{1}}{1-k_{1}}. \end{array}\right. \end{array}$$

#### 3.1.7. $S_{L}\to S_{L}$

The full battery of RS remains its status when it has sufficient energy but decodes the superimposed signal erroneously in Mode II. The amount of remaining energy harvested by RS could be any value in this case, because the amount of battery energy has already reached the upper limit. Therefore, the transition probability is obtained as

(21) $$\begin{array}{cc} P_{L,L} & =\mathrm{Pr}\;\left\{\;\left(\gamma_{br1}\;<\;R_{P}\right)⋃\left[\left(\gamma_{br1}\;\geq \;R_{P}\right)⋂\left(\gamma_{br2}\;<\;R_{s}\right)\right]\;\right\}\\ & =F_{{\left|h_{br}\right|}^{2}}\left(\phi_{1}\right) \end{array}$$

#### 3.1.8. $S_{m}\to S_{l}$

The event occurs only in Mode III, and its corresponding transition probability can be derived as

(22) $$\begin{array}{cc} P_{m,l} & =\mathrm{Pr}\left\{\left(\gamma_{br}^{x_{p}}\geq R_{P}\right)⋂\left(\gamma_{br}^{x_{s}}\geq R_{s}\right)⋂\left(E_{T}=\frac{\left(m-l\right)E_{C}}{L}\right)\right\}\;\\ & =\left\{\begin{array}{c} \exp\left(-\frac{\theta_{1}}{\lambda_{br}}\right),ifE_{T}=\frac{\left(m-l\right)E_{C}}{L};\;\\ 0,ifE_{T}\neq \frac{\left(m-l\right)E_{C}}{L}. \end{array}\right. \end{array}$$

where $\theta_{1}=\max\left(\varphi_{1},\phi_{1}\right)$.

According to the above analysis and the MC properties [23], we can obtain the state transition matrix, i.e., $P={\left[P_{i,j}\right]}_{\left(L+1)(L+1\right)}$, which will be used to obtain a unique steady-state probability vector. The probability vector can be calculated by solving a set of balance equations, as follows:

(23) $$\begin{array}{c} \pi ={\left(B+P^{T}-I\right)}^{-1}b \end{array},$$

where $\pi^{\prime }={\left[\pi_{0}^{\prime },\pi_{1}^{\prime },\cdots ,\pi_{L}^{\prime }\right]}_{1\times (L+1)}^{T}$ and $\sum_{i=0}^{L}\pi_{i}^{\prime }=1$, **I** denotes an identity matrix, **B** represents a matrix with $\forall B_{i,j}=1\left(1\leq i\leq L+1,1\leq j\leq L+1\right)$ and $b={\left(1,1,\ldots ,1\right)}^{T}$ [24]. Hence, the probability that the remaining amount of RS’s energy exceeds or equals $E_{T}$ can be illustrated as

(24) $$P_{e}=\sum_{i=l}^{L}\pi_{i}^{\prime },l=\underset{l\in 1,\cdots ,L}{\arg\min}\left\{E_{l}\geq E_{T}\right\}.$$

### 3.2. Outage Probability with Discrete Time Energy Harvesting

In any transmission block, occurrence probabilities of Mode I, Mode II, and Mode III are, respectively, illustrated as P(A), P(B), and P(C). $P_{out}\left(P_{A}\right)$, $P_{out}\left(P_{B}\right)$, and $P_{out}\left(P_{C}\right)$ are the outage probabilities of the primary system in Mode I, Mode II and Mode III, respectively, while $P_{out}\left(S_{A}\right)$, $P_{out}^{DF}\left(S_{B}\right)$ and $P_{out}^{DF}\left(S_{C}\right)$ represent the secondary system’s outage probabilities of Mode I, Mode II, and Mode III, respectively. According to total probability theory, the outage probabilities of the primary system and secondary system are expressed by

(25) $$\begin{array}{cc} P_{out}\left(P\right)= & P\left(A\right)P_{out}\left(P_{A}\right)+P\left(B\right)P_{out}\left(P_{B}\right)\\ & +P\left(C\right)P_{out}\left(P_{C}\right),\\ P_{out}\left(S\right)= & P\left(A\right)P_{out}\left(S_{A}\right)+P\left(B\right)P_{out}\left(S_{B}\right)\\ & +P\left(C\right)P_{out}\left(S_{C}\right), \end{array}$$

respectively. According to (24), the occurance probability of Mode I equals the probability that the amount of RS energy has not reached the transmission threshold value $E_{T}$, which can be derived as $P\left(A\right)=1-P_{e}.$

Mode II occurs when the energy of RS reaches the transmission threshold but the superimposed signal is erroneously decoded at the RS. Therefore, the occurrence probability can be given by

(26) $$\begin{array}{cc} P\;\left(B\right) & =\;P_{e}\;\mathrm{Pr}\;\left\{\;\left(\gamma_{br1}\;<\;R_{p}\right)\;\cup \;(\;\left(\gamma_{br1}\;\geq \;R_{p}\right)\;\cap \;\left.\;\left(\gamma_{br2}\;<\;R_{s}\right)\;)\;\right.\right\}\\ & =P_{e}F_{{\left|h_{br}\right|}^{2}}\left(\phi_{1}\right) \end{array}$$

Mode III corresponds to a situation where RS not only has enough energy, but also correctly decodes the superimposed signal. Hence, its occurrence probability can be derived as

(27) $$\begin{array}{cc} P\left(C\right) & =P_{e}\mathrm{Pr}\left\{\gamma_{br1}\geq R_{P},\gamma_{br2}\geq R_{s}\right\}\\ & =P_{e}\exp\left(-\frac{\theta_{1}}{\lambda_{br}}\right) \end{array}$$

#### 3.2.1. Outage Probability of Primary System

In Mode I, BS conveys the signal to both PU and SU directly, without RS. The probability that primary system is in outage can be written as

(28) $$\begin{array}{c} P_{out}\left(P_{A}\right)\;=\;\mathrm{Pr}\left\{\gamma_{bp}\;<\;R_{p}\right\}\;=\;\mathrm{Pr}\;\left\{\;|h_{bp}{|}^{2}\;<\;\frac{R_{p}\delta^{2}}{P_{BS}\;\left(k_{1}-\left(1-k_{1}\right)R_{p}\right)\;}\;\right\} \end{array}$$

- If $R_{p}\geq \frac{k_{1}}{1-k_{1}}$,
  (29) $$P_{out}\left(P_{A}\right)=1$$
- If $R_{p}<\frac{k_{1}}{1-k_{1}}$,
  (30) $$P_{out}\left(P_{A}\right)=\mathrm{Pr}\left\{\gamma_{bp}<R_{p}\right\}=1-\exp\left(-\frac{\varphi_{1}}{\lambda_{bp}}\right)$$

In the case of Mode II, RS fails in decoding, so that BS performs a direct transmission. Consequently, the outage probability of the primary system in Mode II is the same as the results of mode I, i.e., $P_{out}\left(P_{B}\right)\;=\;P_{out}\left(P_{A}\right)$.

The event of outage occurs in Mode III when neither direct nor cooperative transmission succeeds. Therefore, the outage probability of the primary system can be illustrated by

- If $R_{p}\geq \frac{k_{1}}{1-k_{1}}$,
  (31) $$\begin{array}{cc} P_{out}\left(P_{C}\right) & =\mathrm{Pr}\left\{\gamma_{bp}<R_{p}\right\}\mathrm{Pr}\left\{\gamma_{rp}<R_{p}\right\}\\ & =1\;-\;\exp\left(\;-\frac{R_{p}\delta^{2}}{P_{RS}k_{2}\lambda_{rp}}\right) \end{array}$$
- If $R_{p}<\frac{k_{1}}{1-k_{1}}$,
  (32) $$\begin{array}{cc} P_{out}\left(P_{C}\right) & =\mathrm{Pr}\left\{\gamma_{bp}<R_{p}\right\}\mathrm{Pr}\left\{\gamma_{rp}<R_{p}\right\}\\ & =\mathrm{Pr}\left\{|h_{bp}{|}^{2}<\varphi_{1}\right\}\mathrm{Pr}\left\{|h_{rp}{|}^{2}<\frac{R_{p}\delta^{2}}{P_{RS}k_{2}}\right\}\\ & =\;\left[1\;-\;\exp\left(\;-\frac{\varphi_{1}}{\lambda_{bp}}\right)\;\right]\;\left[1\;-\;\exp\left(\;-\frac{R_{p}\delta^{2}}{P_{RS}k_{2}\lambda_{rp}}\right)\;\right] \end{array}$$

#### 3.2.2. Outage Probability of Secondary System

The outage probabilities of the secondary system under the proposed relaying scheme are similar to that of primary system. BS directly conveys the signal to both PU and SU without the assistance of RS in Mode I, and the system performs direct transmission in Mode II due to unsuccessful decoding at RS. Therefore, the outage probabilities of SU in Mode I and Mode II are the same and expressed as follows

(33) $$\begin{array}{cc} P_{out}\left(S_{A}\right) & =1-\mathrm{Pr}\{\gamma_{bs1}\geq R_{p},\gamma_{bs2}\geq R_{s}\}\\ & =1-\exp\left(-\frac{\theta_{1}}{\lambda_{bs}}\right), \end{array}$$

(34) $$P_{out}\left(S_{B}\right)=P_{out}\left(S_{A}\right),$$

respectively.

An outage event occurs in Mode III when neither direct nor cooperative transmission succeeds. The SU’s outage probability in Mode III is derived as

(35) $$\begin{array}{cc} P_{out}\left(S_{C}\right) & =\left(1\;-\;\mathrm{Pr}\{\gamma_{bs1}\;\geq \;R_{p},\gamma_{bs2}\;\geq \;R_{s}\}\right)\mathrm{Pr}\left\{\gamma_{rs}\;<\;R_{s}\right\}\\ & =\left[1-\mathrm{Pr}\left\{{\left|h_{bs}\right|}^{2}\geq \varphi_{1},{\left|h_{bs}\right|}^{2}\geq \phi_{1}\right\}\right]\\ & \;\times \mathrm{Pr}\left\{{\left|h_{rs}\right|}^{2}<\frac{R_{s}\delta^{2}}{P_{RS}\left(1-k_{2}\right)}\right\}\\ & =\left[1\;-\;\exp\left(\;-\frac{\theta_{1}}{\lambda_{bs}}\right)\right]\;\left[1\;-\;\exp\left(\;-\frac{R_{s}\delta^{2}}{P_{RS}\left(1-k_{2}\right)\lambda_{rs}}\right)\;\right]. \end{array}$$

### 3.3. Outage Capacity

Outage capacity is defined as the maximum constant rate that can be maintained over fading blocks with a specified outage probability and is used for slowly varying channels where the instantaneous signal-to-noise-radio (SNR) is assumed to be constant [25]. Therefore, the outage capacity of primary system and secondary system $C_{out}\left(P\right)$ and $C_{out}\left(S\right)$ can be expressed as

(36) $$\begin{array}{c} C_{out}\left(P\right)=\left[1-P_{out}\left(P\right)\right]log_{2}\left(1+\gamma_{th}^{P}\right),\\ C_{out}\left(S\right)=\left[1-P_{out}\left(S\right)\right]log_{2}\left(1+\gamma_{th}^{S}\right), \end{array}$$

respectively, where $\gamma_{th}^{p}=R_{p}$ and $\gamma_{th}^{S}=R_{s}$, which are related to $r_{p}$ and $r_{s}$.

## 4. Power Allocation Parameters Optimization

According to the above-mentioned analysis results of the outage probability of the primary system and secondary system, we utilize the above analysis results to calculate the spectrum efficiency $\eta_{SE}$ of the overall system, which can be defined as $\eta_{SE}=r_{p}\left(1-P_{out}\left(P\right)\right)+r_{s}\left(1-P_{out}\left(S\right)\right)$. Then, the energy efficiency (EE) $\eta_{EE}$ of the proposed system can be expressed as $\eta_{EE}=\frac{\eta_{SE}}{P_{BS}}$. Furthermore, the parameter power allocation coefficient $k_{1}$ and $k_{2}$ will affect the spectrum efficiencies and EE of the overall systems. For the proposed cooperative transmission protocol for the considered system, if more transmission power is allocated to transmit the signal of the PU, the outage performance of the primary system will be improved while the achievable rate of the secondary system will decrease. Conversely, if more energy is used to transmit the information of the SU, information transmission of the secondary system will cause greater interference to the information transmission of the primary system. Therefore, obtaining the optimal power allocation is essential to realizing a mutual improvement in performance for both the primary system and secondary system.

Following the above outage performance analyses of the overall system, we need to maximize the SE of the secondary system while ensuring that the SE of the primary system is no less than a given threshold ε, so as to obtain the globally optimal power allocation. Thus, the corresponding optimization problem $\left(OP1\right)$ can be defined as

(37) $$\begin{array}{cc} \max & r_{s}\left(1-P_{\mathrm{out}}^{S}\left(k_{1},k_{2}\right)\right)\\ s.t. & C1:r_{p}\left(1-P_{\mathrm{out}}^{P}\left(k_{1},k_{2}\right)\right)\geq \varepsilon ;\\ & C2:0<k_{1}<1;\\ & C3:0<k_{2}<1. \end{array}$$

From (37), $r_{s}$, $r_{p}$, and ε are the known parameters. Hence, the optimization problem $\left(OP1\right)$ can be converted to $\left(OP2\right)$

(38) $$\begin{array}{cc} \min & P_{\mathrm{out}}^{S}\left(k_{1},k_{2}\right)\\ s.t. & C1:P_{\mathrm{out}}^{P}\left(k_{1},k_{2}\right)\leq 1-\frac{\varepsilon }{r_{p}};\\ & C2:0<k_{1}<1;\\ & C3:0<k_{2}<1. \end{array}$$

According to the analyses of outage probabilities for both the primary and secondary systems, we can see that the system performs direct transmission when $k_{1}\leq \frac{R_{P}}{1+R_{P}}$, which makes the cognitive transmission meaningless. $k_{1}$ must be higher than 0.5, due to the execution of NOMA. Thus, we reset the value range of $k_{1}$ as $\left(max(\frac{R_{P}}{1+R_{P}},0.5),1\right)$. When $k_{1}$ takes a fixed value, the optimization problem (OP2) can be derived as (OP3) by substituting (25)–(33) into (38)

(39) $$\begin{array}{cc} \min & \rho_{1}+\rho_{2}\left[1-\exp(-\frac{R_{s}}{\beta \lambda_{rs}\left(1-k_{2}\right)})\right]\\ s.t. & C1:\rho_{3}+\rho_{4}\left[1-\exp(-\frac{R_{p}}{\beta \lambda_{rp}k_{2}})\right]\leq 1-\frac{\varepsilon }{r_{p}};\\ & C2:0<k_{2}<1; \end{array}$$

where

(40) $$\begin{array}{c} \beta =\frac{P_{RS}}{\delta^{2}}\\ \rho_{1}=\left(1-P_{e}\right)\left[1-\exp\left(-\frac{\theta_{1}}{\lambda_{bs}}\right)\right]+P_{e}F_{{\left|h_{br}\right|}^{2}}\left(\phi_{1}\right)\left[\left[1-\exp\left(-\frac{\theta_{1}}{\lambda_{bs}}\right)\right]\right]\\ \rho_{2}=P_{e}\exp\left(-\frac{\theta_{1}}{\lambda_{br}}\right)\left[1-\exp\left(-\frac{\theta_{1}}{\lambda_{bs}}\right)\right]\\ \rho_{3}=\left(1-P_{e}\right)\left[1-\exp\left(-\frac{\varphi_{1}}{\lambda_{bp}}\right)\right]+P_{e}F_{{\left|h_{br}\right|}^{2}}\left(\phi_{1}\right)\left[1-\exp\left(-\frac{\varphi_{1}}{\lambda_{bp}}\right)\right]\\ \rho_{4}=P_{e}\exp\left(-\frac{\theta_{1}}{\lambda_{br}}\right)\left[1-\exp\left(-\frac{\varphi_{1}}{\lambda_{bp}}\right)\right]. \end{array}$$

Obviously, when $k_{1}$ takes a fixed value, $\rho_{1},\rho_{2},\rho_{3},\rho_{4}$ can be regarded as constants and the subject function decreasing with $k_{2}$ decreases while the constraint function increases. We want to find the minimum value of the subject function while satisfying the constraints function. Therefore, we set $P_{\mathrm{out}}^{P}\left(k_{1}^{*},k_{2}\right)=1-\frac{\varepsilon }{r_{p}}$ and $k_{2}$ can be derived as

(41) $$k_{2}=\frac{\beta \lambda_{rp}}{R_{p}}\ln\left(\frac{r_{p}\rho_{4}}{r_{p}\left(\rho_{3}+\rho_{4}-1\right)+\varepsilon }\right).$$

The subjective function of (OP3) is a convex function, which is because the value of the second derivative of the subject function is the positive number. Hence, (OP3) is a convex optimization problem [26] and we can obtain the globally optimal solution $\left(k_{1}^{*},k_{2}^{*}\right)$ by utilizing a one-dimension search over $k_{1}$ as the following algorithm in Algorithm 1.
**Algorithm 1:** The proposed solution for problem (OP3).  **step1**: Considering the cooperative transmission situation, we defined the value interval of $k_{2}\in \left(0,1\right)$;  **step2**: For the given $k_{1}\in \left(max(\frac{R_{P}}{1+R_{P}},0.5),1\right)$, obtaining the optimal $k_{2}^{*}$ by letting $P_{\mathrm{out}}^{P}\left(k_{1},k_{2}\right)=1-\frac{\varepsilon }{r_{p}}$ and constraint C2 in (41)  **step3**: Define $\Delta k_{1}$ as the search step and update $k_{1}=k_{1}+\Delta k_{1}$ to perform a one-dimension search over $k_{1}$, i.e., repeat Step 1–Step 3;  **step4**: Choose the optimal result from the following equation $\left(k_{1}^{*},k_{2}^{*}\right)=\arg\max problem\left(OP3\right)$


## 5. Numerical Results

**Figure 3:**
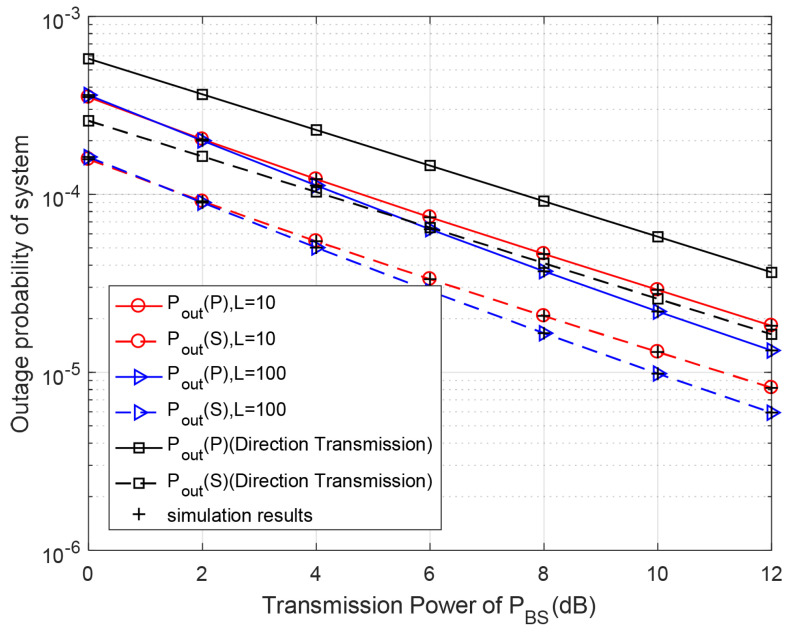
Outage probability with respect to different primary transmission powers $P_{BS}$ for different discrete levels of battery capacity. Primary target rate $r_{p}=0.5$ bps/Hz, secondary target rate $r_{s}=0.5$ bps/Hz, $P_{RS}=P_{BS}$, $k_{2}=k_{1}=0.8$.

In the proposed system model, the BS transmits the superimposed signal to PU and SU with/without the assistance of RS. RS with a battery performs discrete-time energy harvesting for opportunistically cooperative transmission, where the charging and discharging process is modeled as an MC with finite states. Besides this, a joint optimization algorithm of power allocation is considered at BS and RS. In this section, the accuracy of the derived expressions is verified through simulation experiment and the impact of each system’s parameters on the performance of the proposed cooperative transmission protocol is demonstrated by adopting actual values. Unless otherwise specified, the simulation parameters in the this system model are set as Table 1:

Figure 3 depicts the outage probabilities of the systems versus the BS’s transmission power for different discrete levels of battery capacity in proposed DF-relaying protocol. When $P_{BS}$ increases, the outage probabilities of the primary system for different discrete levels of battery capacity gradually become lower and are always smaller than that of the direct transmission scheme. The outage probabilities of both the primary system and secondary system become lower as the numbers of battery levels increase, because it can reduce energy wasting during discretised energy harvesting. The theoretical results are consistent with Monte-Carlo simulations.

**Figure 4:**
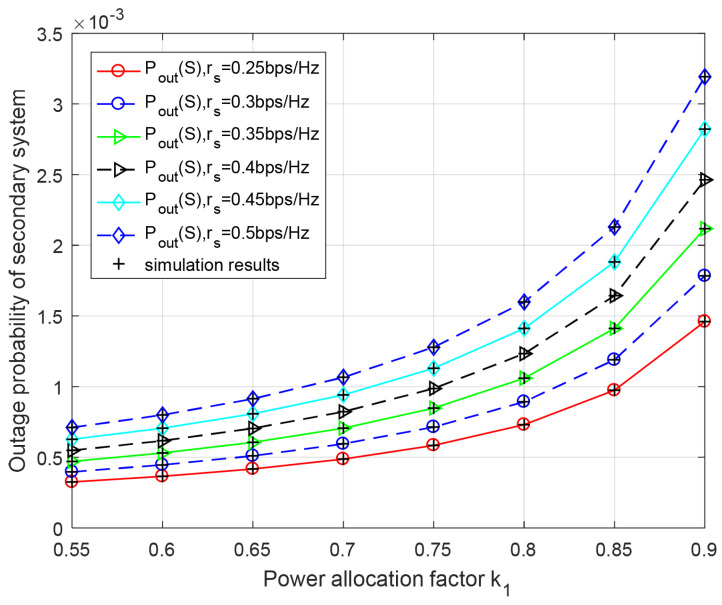
Outage performance of secondary system with respect to power allocation factor $k_{1}$ for different secondary rates $r_{s}$. $k_{2}=k_{1}$.

**Figure 5:**
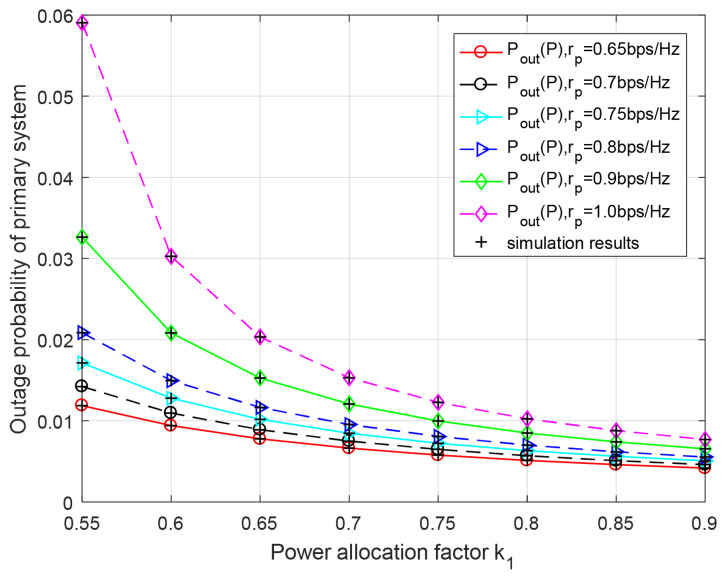
Outage performance of primary system with respect to power allocation factor $k_{1}$ for different primary target rates $r_{p}$. $k_{2}=k_{1}$.

Figure 4 and Figure 5 show secondary and primary outage probabilities versus the power allocation factor $k_{1}$ for different transmission rates $r_{s}$ and $r_{p}$, respectively. From Figure 4, it can be observed that each curve is going up as higher power allocation factor $k_{1}$ increases. Less power is allocated for the secondary data transmission. Moreover, we have also observed that the secondary system’s performance will be deteriorated as $r_{s}$ increases, because the transmission rate supported by the channel is limited for a certain $k_{1}$ and $k_{2}$. Similarly, we know that the outage performance of the primary system improves, while power allocation factor $k_{1}$ increases in Figure 5. Meanwhile, the theoretical results coincide exactly with Monte-Carlo simulation.

**Figure 6:**
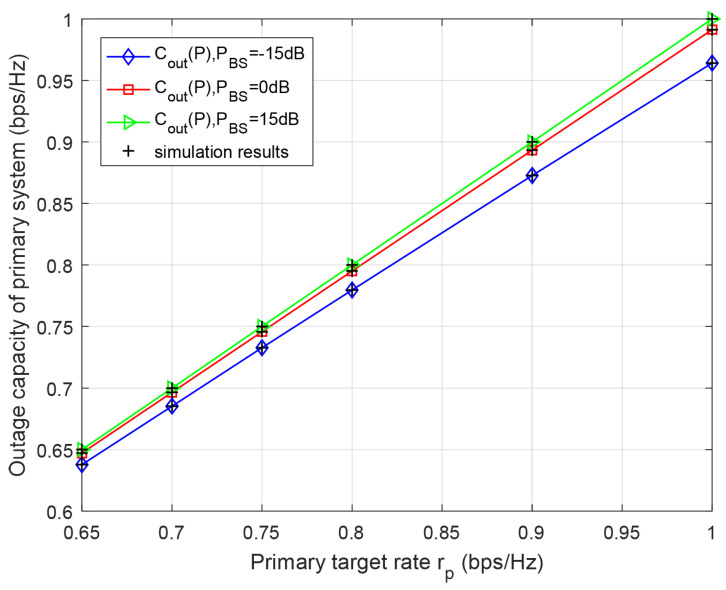
Outage capacity of primary system with respect to primary target rate $r_{p}$ for different BS’s transmission power $P_{BS}$. $P_{RS}=P_{BS}$, $k_{2}=k_{1}=0.8$.

**Figure 7:**
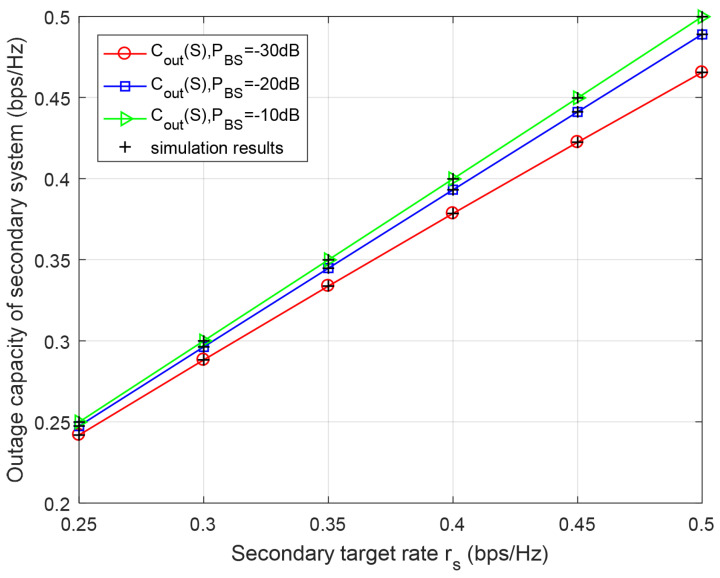
Outage capacity of secondary system with respect to secondary target rate $r_{s}$ for different BS’s transmission power $P_{BS}$. $P_{RS}=P_{BS}$, $k_{2}=k_{1}=0.8$.

Figure 6 and Figure 7 reveal that secondary and primary outage capacities versus primary and secondary target rates $r_{p}$ and $r_{s}$ for different BS’s transmission power $P_{BS}$, respectively. From Figure 6 and Figure 7, it can be seen that the outage capacities of both primary and secondary systems increase with a higher target rate $r_{p}$ and $r_{s}$, which, because of the higher SNR thresholds, can be caused by the increased target rate. Furthermore, with the increase in transmission power of BS for a certain target rate, the outage capacities of the primary and secondary system will correspondingly be increased, since higher transmission power reduces the outage probabilities of primary and secondary systems in Figure 6 and Figure 7. The results also are in good agreement with Monte-Carlo simulation.

**Figure 8:**
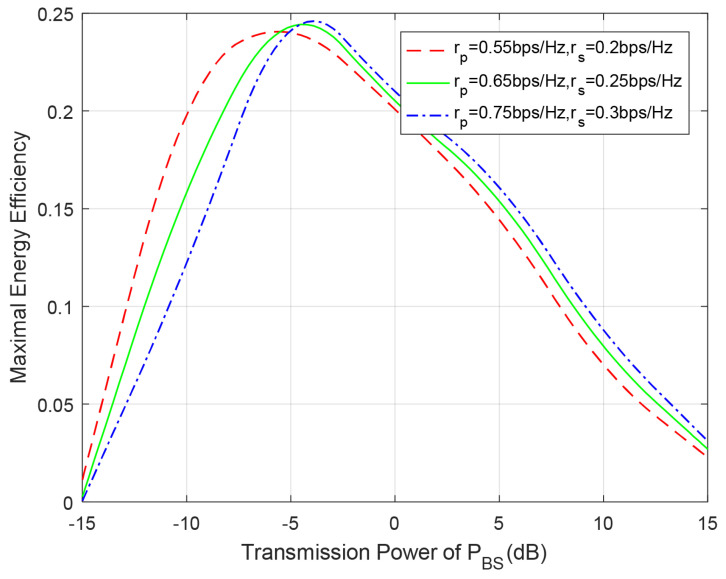
Maximal EE of the whole system with respect to BSs’ transmission power for different transmission rates. $P_{RS}=P_{BS}$.

In the following, we will discuss the EE of the overall system and adopt the optimal algorithm, which are proposed in the above section, in simulations. Figure 8 shows the maximal EE of the overall system with respect to BSs’ transmission power for different transmission rates. It is can be seen from this figure that the value of maximal EE first improves, and then deteriorates with BSs’ transmission power $P_{BS}$ increases. The EE reaches its largest value at around −5 dB. In addition, we can see from this figure that the average EE of the overall system will improve when the transmission rates are higher. This is because the increased outage probability can partly be compensated by the higher transmission rate.

**Figure 9:**
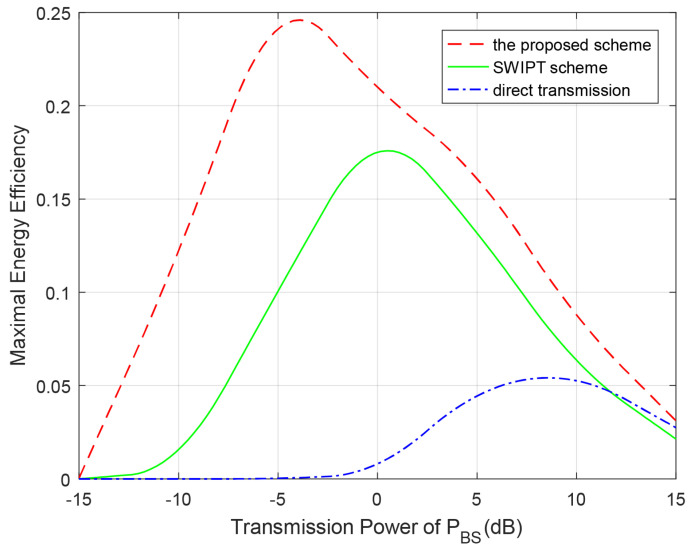
Maximal EE of the overall system with respect to BSs’ transmission power for different spectrum sharing schemes. $P_{RS}=P_{BS}$, $r_{p}=0.75$ bps/Hz, $r_{s}=0.3$ bps/Hz.

Figure 9 shows average EE of the overall system with respect to BSs’ transmission power for different spectrum sharing schemes. We choose the direct transmission scheme and the transmission scheme in [17] as the benchmark for comparison. In [17], the system model is also established in the CR-NOMA network, the system architecture and channel environment are similar as ours. However, they adopted SWIPT technology for EH and information transmission. We can see from the figure that the EE of our scheme, direct transmission scheme and SWIPT scheme in [17] improve as $P_{BS}$ increases. After the value reaches a peak point, the changes become opposite. In addition, our proposed spectrum-sharing scheme obviously outperforms two other schemes in the EE.

## 6. Conclusions

In this paper, we proposed a cooperative transmission protocol in a NOMA-CRN, which improved the spectrum efficiency of the overall system and reduced the interference between PUs and SUs at the same time. To improve the performance of the system and prolong the life of the relay node, we introduced RF energy harvesting for relay node with finite battery. Moreover, an MC model based on discrete-time energy harvesting was developed to analyze the charging and discharging performance of the relay node, and then the analytical expressions of the outage probabilities and the outage capacities for the primary system and the secondary system were derived. Besides, in order to optimize the average EE of the system, we proposed a joint parameter optimization algorithm to obtain optimal power allocation coefficients $k_{1}$ and $k_{2}$. Finally, the correctness of analysis and deduction was verified by Monte Carlo simulation.

On one hand, we revealed that discrete-time EH was beneficial to reducing the outage probability of the proposed NOMA-CRN model in the practical application, especially when the system had a higher number of battery levels. On the other hand, we knew that the power allocation factors $k_{1}$ and $k_{2}$ were essential for improving the performance of the overall system and the proposed joint parameter optimization algorithm realized a mutual improvement in performance for both primary and secondary systems. Besides this, the simulation results showed that the EE of the proposed scheme was better than that of the direct transmission scheme and the transmission scheme based on SWIPT.

## Figures and Tables

**Table 1:** Lists of Necessary Parameters.

Symbol	Name	Value
$d_{bp}$	Transmit distance from BS to PU	10 m
$d_{bs}$	Transmit distance from BS to SU	5 m
$d_{br}$	Transmit distance from BS to RS	2.5 m
$d_{rp}$	Transmit distance from RS to PU	7.5 m
$d_{rs}$	Transmit distance from RS to SU	2.5 m
$\lambda_{i}$	The means of channel gain	$d_{i}^{-3}$(i=bp,br,bs,rp and rs)
η	Energy conversion efficiency	0.5
$E_{c}$	Total capacity of battery at RS	20 dBm
$E_{T}$	Predefined threshold power at RS	−10 dBm
$\delta^{2}$	AWGNs	−30 dBm
*L*	RS’s battery levels	50
$r_{p}$	Primary target rate	0.65 bps/Hz
$r_{s}$	Secondary target rate	0.25 bps/Hz
$P_{BS}$	transmission power of BS	−10 dBm
$P_{RS}$	transmission power of RS	−10 dBm

## Data Availability

Data is contained within the article.

## Appendix A. Derivation of (18)

The Equation (18) can be rewritten as

(A1) $$\begin{array}{cc} P_{l,l}= & \mathrm{Pr}\left\{\;\left[\left(E_{T}>\frac{lE_{C}}{L}\right)⋂\left(E_{H}^{I}<\frac{E_{C}}{L}\right)\right]\right.\\ & \;⋃[\left(E_{T}\leq \frac{lE_{C}}{L}\right)⋂\left(E_{H}^{\mathrm{II}}<\frac{E_{C}}{L}\right)\\ & \;\left.⋂\;\left(\;\left(\gamma_{br1}<R_{P}\right)⋃\;\left(\;\left(\gamma_{br1}\geq R_{P}\right)⋂\left.\left(\gamma_{br2}<R_{s}\right)\right)\right.\;\right)\;]\;\right\} \end{array}$$

When $E_{T}>\frac{lE_{C}}{L}$, the probability can be derived as

(A2) $$P_{l,l}=F_{{\left|h_{br}\right|}^{2}}\left(\frac{E_{C}}{L\eta P_{BS}}\right)$$

When $E_{T}\leq \frac{lE_{C}}{L}$, the corresponding probabilities of each different situation are detailed as follow (see (A3)):

(A3) $$\begin{array}{cc} P_{l,l} & =\mathrm{Pr}\left\{\;\left({\left|h_{br}\right|}^{2}\;<\;\frac{E_{C}}{L\eta P_{BS}}\right)\right.⋂\left[\;\left({\left|h_{br}\right|}^{2}\;<\;\frac{R_{P}\delta^{2}}{P_{BS}k_{1}-P_{BS}\left(1-k_{1}\right)R_{P}}\right)\;⋃\right.\\ & \;\left.\left.\left({\left|h_{br}\right|}^{2}\;\geq \;\frac{R_{P}\delta^{2}}{P_{BS}k_{1}-P_{BS}\left(1-k_{1}\right)R_{P}},{\left|h_{br}\right|}^{2}\;<\;\frac{R_{s}\delta^{2}}{P_{BS}\left(1-k_{1}\right)}\right)\;\right]\;\right\}\\ & =\underset{D_{1}}{\underset{⏝}{\mathrm{Pr}\left\{{\left|h_{br}\right|}^{2}<\frac{E_{C}}{L\eta P_{BS}},{\left|h_{br}\right|}^{2}<\frac{R_{P}\delta^{2}}{P_{BS}k_{1}-P_{BS}\left(1-k_{1}\right)R_{P}}\right\}}}\\ & ⋃\underset{D_{2}}{\underset{⏝}{\mathrm{Pr}\left\{{\left|h_{br}\right|}^{2}<\frac{E_{C}}{L\eta P_{BS}},{\left|h_{br}\right|}^{2}\geq \frac{R_{P}\delta^{2}}{P_{BS}k_{1}-P_{BS}\left(1-k_{1}\right)R_{P}},{\left|h_{br}\right|}^{2}<\frac{R_{s}\delta^{2}}{P_{BS}\left(1-k_{1}\right)}\right\}}} \end{array}$$

For the first term $D_{1}$,

(1) $R_{P}\leq \frac{E_{C}k_{1}}{\delta^{2}L\eta +E_{C}\left(1-k_{1}\right)}<\frac{k_{1}}{1-k_{1}}$,
  (A4) $$P_{l,l}^{II(a1)}=F_{{\left|h_{br}\right|}^{2}}\left(\frac{R_{P}\delta^{2}}{P_{BS}k_{1}-P_{BS}\left(1-k_{1}\right)R_{P}}\right)$$
(2) $R_{P}>\frac{E_{C}k_{1}}{\delta^{2}L\eta +E_{C}\left(1-k_{1}\right)}$,
  - If $R_{P}<\frac{k_{1}}{1-k_{1}}$,
    (A5) $$P_{l,l}^{II(a2)}=F_{{\left|h_{br}\right|}^{2}}\left(\frac{E_{C}}{L\eta P_{BS}}\right)$$
  - If $R_{P}\geq \frac{k_{1}}{1-k_{1}}$,
    (A6) $$P_{l,l}^{II(a3)}=0$$

For the first term $D_{2}$,

(1) $R_{s}\leq \frac{E_{C}\left(1-k_{1}\right)}{\delta^{2}L\eta }$
  - If $R_{P}<\frac{k_{1}}{1-k_{1}}$,
    (A7) $$P_{l,l}^{II(b1)}=F_{{\left|h_{br}\right|}^{2}}\left(\frac{R_{s}\delta^{2}}{P_{BS}\left(1-k_{1}\right)}\right)-F_{{\left|h_{br}\right|}^{2}}\left(\frac{R_{P}\delta^{2}}{P_{BS}k_{1}-P_{BS}\left(1-k_{1}\right)R_{P}}\right)$$
  - If $R_{P}\geq \frac{k_{1}}{1-k_{1}}$,
    (A8) $$P_{l,l}^{II(b2)}=F_{{\left|h_{br}\right|}^{2}}\left(\frac{R_{s}\delta^{2}}{P_{BS}\left(1-k_{1}\right)}\right).$$
(2) $R_{s}>\frac{E_{C}\left(1-k_{1}\right)}{\delta^{2}L\eta }$
  - If $R_{P}<\frac{k_{1}}{1-k_{1}}$,
    (A9) $$P_{l,l}^{II(b3)}\;=\;F_{{\;\left|h_{br}\;\right|}^{2}}\;\left(\;\frac{E_{C}}{L\eta P_{BS}}\;\right)\;-\;F_{{\;\left|h_{br}\;\right|}^{2}}\;\left(\frac{R_{P}\delta^{2}}{P_{BS}k_{1}\;-\;P_{BS}\left(1-k_{1}\right)R_{P}}\;\right)$$
  - If $R_{P}\geq \frac{k_{1}}{1-k_{1}}$,
    (A10) $$P_{l,l}^{II(b4)}=F_{{\left|h_{br}\right|}^{2}}\left(\frac{E_{C}}{L\eta P_{BS}}\right)$$

To sum up, the transmission probability of $S_{l}\to S_{l}$ can be concluded as (see (18)).
